# Activation of TrkB/Akt signaling by a TrkB receptor agonist improves long-term histological and functional outcomes in experimental intracerebral hemorrhage

**DOI:** 10.1186/s12929-019-0543-8

**Published:** 2019-07-15

**Authors:** Chun-Hu Wu, Chien-Cheng Chen, Tai-Ho Hung, Yen-Chieh Chuang, Min Chao, Song-Kun Shyue, Szu-Fu Chen

**Affiliations:** 1 Graduate Institute of Life Sciences, National Defense Medical Center, Taipei, Taiwan; 2 Department of Physical Medicine and Rehabilitation, Cheng Hsin General Hospital, 45 Cheng Hsin Street, Taipei, Taiwan, Republic of China; 3 Graduate Institute of Gerontology and Health Care Management, Chang Gung University of Science and Technology, Taoyuan, Taiwan; 4 Department of Obstetrics and Gynecology, Chang Gung Memorial Hospital at Taipei and College of Medicine, Chang Gung University, Taoyuan, Taiwan; 5 Department of Physiology and Biophysics, National Defense Medical Center, Taipei, Taiwan; 6 School of Medicine, National Defense Medical Center, Taipei, Taiwan; 7 Institute of Biomedical Sciences, Academia Sinica, 128 Academia Road, Section 2, Nankang, Taipei, Taiwan, Republic of China

**Keywords:** 7,8-dihydroxyflavone, Apoptosis, Akt, Intracerebral hemorrhage, TrkB

## Abstract

**Background:**

Intracerebral hemorrhage (ICH) induces a complex sequence of apoptotic cascades that contribute to secondary neuronal damage. Tropomyosin-related kinase receptor B (TrkB) signaling plays a crucial role in promoting neuronal survival following brain damage.

**Methods:**

The present study investigated the protective effects and underlying mechanisms of TrkB activation by the specific TrkB agonist, 7,8-dihydroxyflavone (7,8-DHF), in a model of collagenase-induced ICH and in neuronal cultures. Mice subjected to collagenase-induced ICH were intraperitoneally injected with either 7,8-DHF or vehicle 10 min after ICH and, subsequently, daily for 3 days. Behavioral studies, brain edema measurement, and histological analysis were conducted. Levels of TrkB signaling-related molecules and apoptosis-related proteins were analyzed by western blots.

**Results:**

Treatment with 20 mg/kg 7,8-DHF significantly improved functional recovery and reduced brain damage up to 28 days post-ICH. Reduction in neuronal death, apoptosis, and brain edema were also observed in response to 7,8-DHF treatment at 3 days post-ICH. These changes were accompanied by a significant increase in the phosphorylation of TrkB and Akt (Ser473/Thr308) at 1 and 3 days, but had no effect on Erk 44/42 phosphorylation. 7,8-DHF also enhanced the phosphorylation of Ask-1 Ser967 and FOXO-1, downstream targets of Akt at 1 and 3 days. Moreover, 7,8-DHF increased brain-derived neurotrophic factor levels at 1 day. In primary cultured neurons stimulated with hemin, 7,8-DHF promoted survival and reduced apoptosis. Furthermore, delaying the administration of 7,8-DHF to 3 h post-ICH reduced brain tissue damage and neuronal death.

**Conclusions:**

Our findings demonstrate that the activation of TrkB signaling by 7,8-DHF protects against ICH via the Akt, but not the Erk, pathway. These data provide new insights into the role of TrkB signaling deficit in the pathophysiology of ICH and highlight TrkB/Akt as possible therapeutic targets in this disease.

## Background

Intracerebral hemorrhage (ICH) is the second most common subtype of stroke with high mortality and morbidity [1]. The current therapeutic interventions focus mainly on supportive care and surgery. So far, no neuroprotective agents have shown clinical benefits in large phase II/III randomized controlled trials, despite their protective effect in animal hemorrhagic stroke model [2]. Thus, the development of effective pharmacological treatment for ICH is urgently needed. Following ICH, a key process contributing to secondary neuronal loss at the periphery of the hematoma is the apoptotic event. Clinical studies have demonstrated the presence of apoptotic cells in brains following ICH [3], and serum caspase-3 levels correlated with severity and outcome in ICH patients [4]. Experimentally, neuroprotective agents targeting apoptosis-related molecules protected against ICH [5, 6]. These studies indicate that apoptosis could serve as a therapeutic target following ICH.

ICH triggers two central pathways of apoptosis: the “extrinsic” pathway occurring through extracellular ligands binding to cell surface death receptors, or the “intrinsic” pathway induced by intracellular signals [7]. Intrinsic apoptosis is caused by the release of mitochondrial intermembrane space proteins, such as cytochrome C (CytoC) and Smac/DIABLO, into the cytosol, leading to subsequent caspase activation. The imbalance of proapoptotic Bcl-2 family proteins (e.g., Bax) and antiapoptotic Bcl-2 family proteins (e.g., Bcl-2) is considered to be the main mechanism contributing to mitochondrial dysfunction [8]. The “extrinsic pathway” is triggered by ligands binding to cell surface “death receptors” (tumor necrosis factor [TNF] receptor and Fas receptor) [7]. Activated death receptors form complexes with intracellular signaling molecules and procaspase-8, resulting in procaspase-8 auto-cleavage and activation. This also initiates a caspase cascade by directly cleaving effector caspase-3. Other upstream modulators of apoptosis are apoptosis signal-regulating kinase 1 (Ask-1), FOXO-1 and *X*-*linked* inhibitor of apoptosis *protein* (XIAP), which are all downstream targets of Akt. Ask-1 is a member of the mitogen-activated protein kinase kinase kinase (MAP3K) family that induces apoptosis via activating downstream MAP kinases (MAPKs), c-Jun N-terminal kinases (JNKs) and p38 MAPKs [9]. FOXO-1 mediates apoptosis through promoting the transcription of genes involved in both intrinsic and extrinsic apoptosis [10]. XIAP inhibits apoptosis by binding directly to caspase-3, 7, and 9, thereby masking their active sites [11]. The interaction of above molecular pathways may lead to further neuronal injuries following ICH.

Additionally, the hemorrhagic brain also triggers self-protective mechanisms to mitigate tissue damage and promote neuronal survival. Brain-derived neurotrophic factor (BDNF) is a neurotrophin that plays a crucial role in promoting neuronal survival by specifically binding to tropomyosin-related kinase receptor B (TrkB) [12]. This binding results in the autophosphorylation and dimerization of the TrkB receptor, which triggers the activation of downstream phosphatidylinositol 3-kinase (PI3K)/Akt, MAPK/Erk, or PLC-γ survival signaling. The PI3K/Akt signaling pathway is the major TrkB-mediated survival pathway that promotes neuronal survival and protects against apoptosis. Activated Akt maintains mitochondrial integrity by antagonizing pro-apoptotic actions of the Bcl-2 family members, Bad and Bax [13]. Furthermore, activated Akt blocks Fas ligand transcription by phosphorylating FOXO, thereby inhibiting ligand-induced extrinsic apoptosis [14]. However, evidence shows that ICH induces a decrease in BDNF expression [15], implying that this endogenous protective response to counteract neuronal damage is often attenuated. In view of the role of the BDNF-TrkB signaling in neuronal survival, targeting the BDNF-TrkB system may be a therapeutic strategy to reduce ICH-induced brain injury. Systemic administration of BDNF is impractical because BDNF has a brief half-life, large molecular size, and poor blood-brain barrier (BBB) penetration [16]. This obstacle can be overcome by using TrkB agonists that can pass the brain. As a member of flavonoid family compounds, 7,8-Dihydroxyflavone (7,8-DHF) has recently been identified as a specific TrkB agonist which activates its downstream PI3K/Akt and Erk signaling cascades, and crosses the BBB after peripheral administration. In vitro studies have shown that 7,8-DHF protects against cellular apoptosis induced by various stimuli, such as glutamate [17] and H_2_O_2_ [18], and mechanisms mimicking secondary injury cascades in ICH. Administration of 7,8-DHF also enhances the activation of phosphorylated TrkB in the brain [17], and has been shown to exert therapeutic effects in various animal disease models such as ischemic stroke [17], traumatic brain injury [19] and Alzheimer’s disease [20] that are related to deficient BDNF signaling. Nevertheless, the therapeutic efficacy of 7,8-DHF against ICH has not been established. Considering the important role of BDNF/TrkB signaling in neuronal survival in damaged brains, it is possible that employing the selective TrkB agonist 7,8-DHF to act upon this system could positively impact the hemorrhagic brain.

Our aim in the present study was to determine whether the activation of TrkB signaling by 7,8-DHF is protective against ICH in both in vivo and in vitro ICH models. We further examined whether 7,8-DHF can promote the TrkB downstream survival PI3K/Akt or Erk pathway, thereby reducing neuronal damage.

## Materials and methods

### Animals

All animal protocols were carried out according to the Guide for the Care and Use of Laboratory Animals published by the US National Institutes of Health (NIH Publication No. 85–23, revised 1996). Male C57BL/6 mice (age 8–12 weeks, weight 22–28 g) were housed under conditions of controlled temperature (22–25 °C) and humidity (40–60%) with a 12-h/12-h light-dark cycle, and were allowed free access to water and food.

### Experimental protocol

A total of 224 mice were used. Mice that had neurological deficit scores greater than 15 or less than 3 at 3 h post-ICH were excluded from the study. Forty-six mice were excluded due to neurological deficit standards or death after ICH (vehicle: 22/90; DHF20; 5/23, DHF40; 19/87). Twenty-four additional sham-operated control mice were used for metabolic characteristics, histology examination and biochemical assays.

The animals were randomized to different treatment groups by using computer-generated random numbers. All outcome measurements and analyses were performed in a blinded manner. Three studies were conducted. The first study was to determine the optimal dose of 7,8-DHF. Following ICH, animals were randomized into 3 groups: 1) ICH + vehicle, 2) ICH + 20 mg/kg 7,8-DHF (DHF20), and 3) ICH + 40 mg/kg 7,8-DHF (DHF40). Next, 7,8-DHF (TCI America, Portland, OR, USA) dissolved in 60% DMSO (0.1 ml) or a corresponding volume of vehicle (60% DMSO) was administered intraperitoneally (ip) 10 min after ICH and subsequently daily for 3 days (10 min, 24 h, 48 h, and 72 h), and behavior tests (*n* = 12/group) were evaluated as the main outcomes. The dose and route of 7,8-DHF were selected based on our previous work on experimental traumatic brain injury [19]. The 4-dose regimen was chosen because the previous study showed that apoptosis-related signals last for over 3 days after ICH [21]. The results of the first study showed that 40 mg/kg DHF produced more protection against behavior deficits than 20 mg/kg. Therefore, a dose of 40 mg/kg was chosen for all subsequent histology and biochemical experiments.

The second study was to evaluate the effect of DHF40 on brain edema, histological damage, apoptosis, and TrkB-related signals. Testing was as follows: 1) blood biochemistry and histology at day 3 or day 28 (*n* = 6/group); 2) brain water content and hemoglobin assay at day 3 (*n* = 6–7/group), and 3) western blot analysis and enzyme-linked immunosorbent assay (ELISA) at day 1 and day 3 (*n* = 5–7/group). The third study was to investigate the therapeutic potential of delayed administration of 7,8-DHF after ICH. Either DHF40 or vehicle was administered ip at 3 h following ICH and subsequently daily for 3 days (3, 27, 51 and 75 h), and protective effects were assessed using cresyl violet staining and Fluoro-Jade B (FJB) histology (*n* = 7/group).

### Intracerebral hemorrhage model

ICH model was according to a previous study [22]. Briefly, mice were anesthetized with sodium pentobarbital (65 mg/kg, ip; Rhone Merieux, Harlow, UK) and placed into a stereotaxic frame. After retracting the scalp, a Hamilton syringe with a 30-gauge needle was implanted through a 1-mm-diameter burr hole into the right striatum (stereotaxic coordinates: 0.8 mm anterior and 2.5 mm lateral to bregma, 2.5 mm in depth). Bacterial collagenase (0.0375 U in 1 μL of saline; type VII-S; Sigma-Aldrich, St. Louis, MO, USA) was infused into the brain at a rate of 0.1 μL/min over 10 min with an infusion pump to induce ICH, and the needle was left in place for an additional 20 min to prevent reflux. After surgery, the craniotomy was sealed with dental cement to close the scalp. Mice were placed on a heated pad throughout the surgery and recovery period to maintain body temperature at 37.0 ± 0.5 °C. Sham-operated mice received an equal volume of normal saline in the same manner.

### Metabolic characteristics assessment

Following terminal anesthesia, venous blood was collected via direct right atrial puncture. The obtained blood was centrifuged (3500 rpm for 5 min), and the serum was stored at − 20 °C until analysis. Serum blood urea nitrogen (BUN), creatinine (CRE), alanine aminotransferase (ALT), were measured by a chemistry autoanalyzer (Synchron Clinical System LX20; Beckman Coulter, Fullerton, CA) to assess renal and liver functions. Hematological determinations were performed using an automatic hematology analyzer ADVIA 2120i (Siemens, Germany). Total white blood cell (WBC), red blood cell (RBC), hemoglobin (HGB), and platelet (PLT) were determined.

### Behavioral assessments

Behavioral testing was performed before ICH and at 1, 4, 7, 14, 21, and 28 days post-ICH. Animals were pre-trained for 3 days for both the rotarod and beam walking tests.

### Modified neurological severity score

The modified neurological severity score (mNSS) included sensory, reflex, motor and balance tests. The neurological function was scored on a scale of 0–18. One point was given for the inability to perform each test or for absence of a testing reflex.

### Rotarod

The speed of an accelerating rotarod was gradually increased from 6 rpm to 42 rpm within 7 min to observe motor function and balance; the time for mice to fall off was recorded.

### Beam walking

The test was used to assess fine motor coordination and function by measuring the ability of the animals to cross an elevated beam. The time for mice to traverse the beam (not to exceed 60 s) and the hindlimb performance as it crossed the beam (based on a 1 to 7 rating scale) were recorded. A score of 7 was given when animals traversed the beam with two or fewer footslips; 6 was given when animals traversed the beam with less than 50% footslips; 5 was given when animals traversed the beam for more than 50% but less than 100% footslips; 4 was given when animals traversed the beam for 100% footslips; 3 was given for traversal with the affected limb extended and not reaching the surface of the beam; 2 was given when the animal was able to balance on the beam but not traverse it; 1 was given when the animal could not balance on the beam.

### Brain water content

Brain edema was examined by measuring brain water content using the wet-dry/wet brain weight method [23]. After decapitation (under anesthesia), the brains were immediately removed and divided into five parts, consisting of the ipsilateral and contralateral cortex, ipsilateral and contralateral basal ganglia and the cerebellum (which served as an internal control). The samples were weighed (wet weight), then baked at 100 °C for 24 h and reweighed (dry weight). Water content was determined as [(wet weight-dry weight)/wet weight] × 100%.

### Hemoglobin assay

The hemoglobin content of brains that had undergone ICH were quantified using a spectrophotometric assay according to previously described methods [23]. Mice were transcardially perfused and the ipsilateral striatum regions were collected following ICH. Distilled water (300 μL) was added to the hemorrhagic hemisphere, followed by homogenization for 30 s and sonication on ice for 1 min. After centrifugation at 13,000 rpm for 30 min, 60 μL of supernatant was reacted with Drabkin’s reagent (240 μL; Sigma-Aldrich) for 15 min at room temperature. Optical density was then measured at a wavelength of 545 nm to assess the concentration of cyanmethemoglobin. To generate a standard curve, blood was collected by cardiac punctures from anesthetized control mice. Incremental volumes of this blood (0, 0.5, 1.0, 2.0, 4.0, and 8.0 μL) were then added to 300 μL of tissue lysate from a normal hemispheric sample.

### Tissue processing and histology

Following terminal anesthesia, mice were transcardially perfused with PBS followed by 4% paraformaldehyde. Brains were removed, post-fixed in 4% paraformaldehyde overnight, cryoprotected with 30% sucrose, and then sectioned coronally (10 μm) from the level of the olfactory bulbs to the visual cortex.

### Fluoro-jade B staining

Fluoro-Jade B (FJB; Chemicon, Temecula, CA, USA) is a polyanionic fluorescein derivative that binds with high sensitivity and specificity to degenerating neurons. Briefly, sections were rehydrated in graded ethanol solutions (100 and 70%, 5 min each) and distilled water, incubated in 0.06% KMnO_4_ for 30 min, rinsed in distilled water for 2 min, incubated in a 0.001% solution of FJB for 30 min, and observed under a fluorescence microscope (Olympus BX-51; Olympus, Tokyo, Japan) at 450–490 nm.

### TUNEL assay

Terminal deoxynucleotidyl transferase dUTP nick end labeling (TUNEL) assay is used for the detection of fragmented DNA by labeling it with fluorescein isothiocyanate (In situ Cell Death Detection Kit; Roche Molecular Biochemicals, Mannheim, Germany). Sections were incubated in TUNEL reaction mixture containing terminal deoxynucleotidyl transferase (TdT) for 60 min at 37 °C. Sections were then observed and photographed under a fluorescence microscope (Olympus BX-51) with blue (450~490 nm) excitation light. Negative controls were prepared by omission of the enzyme TdT.

### Immunofluorescence staining

1) To assess the cellular source of TrkB, double immunofluorescence labeling was performed by simultaneous incubation of sections with rabbit anti-TrkB (1:100; Cell Signaling Danvers, MA, USA) overnight at 4 °C with mouse anti-NeuN (a neuronal marker; 1:100; Millipore, Billerica, MA, USA), rat anti-GFAP (an astrocyte marker; 1:200; Invitrogen, Camarillo, CA, USA), and mouse anti-F4/80 (a microglia/macrophage marker; 1:100; Serotec, Düsseldorf, Germany).

2) To assess protein expression of pTrkB, pAkt Ser473 or BDNF, double immunofluorescence labeling was performed by simultaneous incubation of sections with rabbit anti-pTrkB (1:100; Cell Signaling), rabbit anti-pAkt Ser473 (1:100; Cell Signaling) or rabbit anti-BDNF (1:100; Santa Cruz, CA, USA) overnight at 4 °C with mouse anti-NeuN (1:100; Millipore) or rat anti-GFAP (1:200; Invitrogen).

Sections were washed, followed by incubation with Alexa Fluor 488- or Alexa Fluor 594-conjugated secondary antibodies (1:500; Molecular Probes, Eugene, OR, USA) for 2 h.

### Injury volume and hemispheric enlargement assessment

Injury volume, hemispheric atrophy, and hemispheric enlargement ratios were quantified using coronal sections stained with cresyl violet at 20 rostral-caudal levels that were spaced 200 μm apart. Sections were analyzed using ImageJ software version 1.50i (ImageJ, National Institutes of Health, Bethesda, MD, USA). Volume measurement was computed by a summation of the areas multiplied by the interslice distance (200 μm). Hemispheric atrophy was assessed using the following formula: ([Contralateral hemisphere or striatal volume − ipsilateral hemisphere or striatal volume]/contralateral hemisphere or striatal volume) × 100%. Hemispheric enlargement was assessed using the following formula: ([ipsilateral hemisphere volume − contralateral hemisphere volume]/contralateral hemisphere volume) × 100%. Analysis was performed by two experimenters who were blinded to all animal groups. Inter-rater reliability was within 10%.

### Quantification of FJB, TUNEL and co-localization staining

FJB, TUNEL assay and double immunofluorescence were quantified on three consecutive sections from the hemorrhagic core at a level of 0.24 mm from the bregma. The number of positive cells was counted in an area of 920 × 860 μm^2^ in 10–12 non-overlapping fields immediately adjacent to the hematoma using a magnification of 200× as previously described [24]. The total number of FJB-positive cells, pTrkB-positive neurons, pAkt Ser473-positive neurons, BDNF-positive neurons or BDNF-positive astrocytes were expressed as the mean number per field of view. Quantification of TUNEL staining was expressed as (TUNEL-stained nuclei/ DAPI-stained nuclei) × 100%. Analysis was performed by two experimenters who were blinded to all animal groups. Inter-rater reliability was within 10%.

### Western blotting

Western blot analysis was performed as previously described [23]. A 3–5-mm coronal section from the injured hemisphere was collected following ICH or sham surgery. Primary neuron cultures were collected at 3 h or 24 h after hemin-induced injury. All samples were centrifuged at 14,000 g for 30 min, and supernatants were used for further protein analysis. Protein concentration was determined by Bradford reagent at 595 nm. Protein samples were denatured in gel-loading buffer at 100 °C for 5 min, separated by electrophoresis on sodium dodecyl sulfate-polyacrylamide gels, and transferred to Immobilon-P membranes (Millipore). Membranes were blocked with 5% milk in PBS-XT and probed overnight at 4 °C with primary antibodies including rabbit anti- cleaved caspase-3 (cCP-3, 1:1000), rabbit anti-pTrkB (1:1000), rabbit anti-pAkt Ser473 (1:1000), rabbit anti-pAkt Thr308 (1:1000), rabbit anti-total Akt (1:1000), rabbit anti-p-extracellular signal-regulated kinases (pErk 44/42; 1:1000), rabbit anti-total Erk (1:2000), rabbit anti-pAsk-1 (1:1000), rabbit anti-total Ask-1 (1:1000) and rabbit anti-pFOXO-1 (1:1000) from Cell Signaling; rabbit anti-total trkB (1:1000), rabbit anti-Bcl-2 (1:1000) and rabbit anti-Bax (1:1000) from Santa Cruz; rabbit anti-Smac/DIABLO (1:500) and rabbit anti-VDAC (1:1000) from Abcam (Cambridge, MA, USA); mouse anti-XIAP (1:1000) and rabbit anti-Cytochrome C (CytoC; 1:1000) from BD Biosciences (San Jose, CA, USA); and mouse anti-β-actin (1:10,000, Sigma-Aldrich).

### Isolation of mitochondria

Dissected hemispheres (prepared as in western blot analysis) were immediately homogenized in 300 μL of ice-cold cytosol extraction buffer (Cytosol/Mitochondria Fractionation kit; Merck, Rockland, Massachusetts, USA) with a protease inhibitor cocktail and DTT. The homogenates were then centrifuged at 700 g for 10 min at 4 °C, and the supernatant was further centrifuged at 10000 g for 30 min at 4 °C. The supernatant obtained after centrifugation at 10000 g was collected as the cytosolic fraction, and the pellet contained the mitochondria. The pellet was resuspended in 50 μL mitochondrial extraction buffer mix containing protease inhibitors and DTT for 10 s and saved as mitochondrial fraction, or maintained intact in PBS at − 80 °C until use.

### ELISA

A 3–5-mm coronal section was taken from the injured hemisphere or sham animals post-ICH. BDNF was measured in brain homogenates using a commercially available ELISA kit (KA0331, Abnova, Walnut, CA, USA).

### Primary cortical neuron cultures, cell viability and cytotoxicity assessment

All media supplies for culture work were purchased from Invitrogen. Primary neuronal cultures were prepared from embryonic C57BL/6 mice at day 15.5 as previously described. Cortical-striatal tissue from 8 to 10 embryos were isolated and digested in 0.5 mg/mL papain, dissociated in Hibernate-A medium (containing B27 supplement), and cultured on 6-well plates at a density of 1 × 10^6^ cells/well. Cultures were maintained in Neurobasal medium supplemented with B27, 10 units/mL penicillin, 10 mg/mL streptomycin, and 0.5 mg/ml glutamine. Three days after plating, arabinofuranosyl cytidine was added to inhibit proliferation of glial cells, and half of the medium was removed and replaced with fresh medium at 4 days. The cells were incubated at 37 °C in an atmosphere containing 10% O_2_, 85% N_2_, and 5% CO_2_ and neurons were used at day 10 in vitro. The purity of neurons was 95% as determined by NeuN immunohistochemical staining. Primary neurons were treated with 10 μM hemin or co-treated with 7,8-DHF and hemin for 24 h and then analyzed by following assessments. Cell viability and cytotoxicity were assessed 24 h post-injury using the 3-[4,5-dimethyl-2-thiazolyl]-2,5-diphenyl-2-tetrazolium bromide (MTT) reduction assay (Sigma-Aldrich; St. Louis, MO) and lactate dehydrogenase (LDH) release assay (LDH assay kit; Roche), respectively. Cells were incubated at 37 °C for 2 h with MTT (0.5 mg/mL; Sigma-Aldrich), and then a solution of anhydrous isopropanol, HCl (0.1 N), and 0.1% Triton X-100 was added to dissolve the water-insoluble formazan. The optical density was determined at 570 nm. Cell viability was expressed as a percentage of the control culture. LDH release was used to quantify cytotoxicity in cultured neurons. Culture supernatants were collected, incubated with substrate mixtures, allowed to undergo a coupled enzymatic reaction that results in the conversion of iodonitrotetrazolium to formazan, and assessed spectrophotometrically for LDH activity at 500 nm. Percent cytotoxicity was calculated by subtracting LDH content in injured cells from total LDH in undamaged controls.

The experiments were repeated 4 times with different batches of primary cultures.

### Statistics

Values are expressed as mean with standard error of the mean (mean ± SEM). Student’s t-test was used to evaluate the difference between two groups. One-way or two-way analysis of variance (ANOVA) followed by post-hoc Bonferroni t-test was used for multiple groups to determine significant differences. Statistical significance was set at *P* < 0.05.

## Results

### 7,8-DHF improved long-term neurobehavioral function and reduced brain edema but did not alter hemorrhage size in mice after ICH

**Fig. 1:**
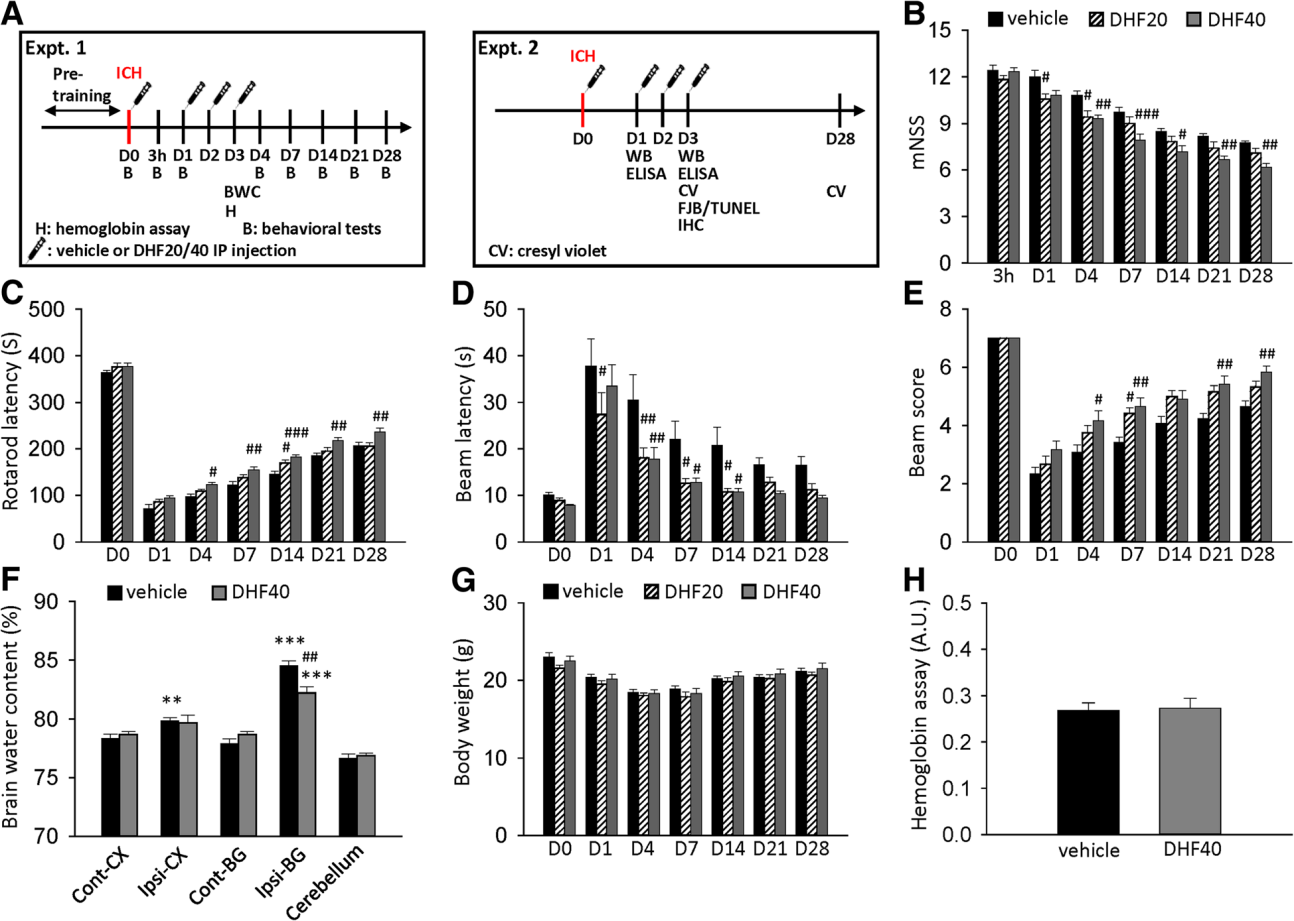
Activation of TrkB by 7,8-DHF treatment improved long-term functional outcomes and reduced brain edema after ICH. **a** The experimental design scheme used to study the effect of TrkB activation by 7,8-dihydroxyflavone in mouse ICH. H: hemoglobin assay. B: behavioral tests. BWC: brain water content. WB: western blots. ELISA: enzyme-linked immunosorbent assay. CV: cresyl violet. FJB: Fluoro-Jade B. TUNEL: Terminal deoxynucleotidyl transferase dUTP nick end labeling. IHC: immunohistochemistry. Behavioral assessment included **b** mNSS, **c** rotarod latency, **d** beam latency, and **e** beam score. Values are mean ± S.E.M; ^#^*P* < 0.05, ^##^*P* < 0.01 and ^###^*P* < 0.001 vs. vehicle group. (*n* = 12 mice / group, two-way ANOVA). **f** Brain edema was assessed by brain water content at 3 days post-ICH. Cont-BG: contralateral basal ganglia; Cont-CX: contralateral cortex; Ipsi-BG: ipsilateral basal ganglia; Ipsi-CX: ipsilateral cortex. Values are mean ± S.E.M; ^**^P < 0.01 and ^***^P < 0.001 vs. contralateral hemisphere; ^##^*P* < 0.01 vs. vehicle group. (*n* = 7 mice / group, Student’s t-test) (**g**) Body weights were recorded up to 28 days post-ICH. Values are mean ± S.E.M; (*n* = 12 mice / group). **h** Hemorrhage size was assessed by hemoglobin assay at 3 days post-ICH. Values are mean ± S.E.M. (*n* = 6 mice / group, Student’s t-test) A.U.: arbitrary units; DHF20: 20 mg/kg 7,8-dihydroxyflavone; DHF40: 40 mg/kg 7,8-dihydroxyflavone

To investigate the protective efficacy of DHF for ICH, we first evaluated motor and neurological functions, which are affected in many ICH patients who are dependent in ambulation and self-care [25]. The extent of global neurological deficit was evaluated by mNSS. At 3 h after injury, there was no difference in mNSS among groups treated with vehicle, DHF20, and DHF40, indicating that the severity of injury was initially similar regardless of treatment (Fig. 1b). Significant improvement in neurological function was observed at 1 and 4 days in the DHF20 group and from 4 to 28 days in the DHF40 group compared to the vehicle group (all *P* < 0.05; Fig. 1b). ICH induced a significant impairment in both rotarod and beam walk performance at all tested time points in the vehicle-treated mice (Figs. 1c-e). Treatment with DHF40 significantly increased the rotarod running time from 4 to 28 days post-ICH as compared to vehicle-treated mice (all *P* < 0.05; Fig. 1c). Similarly, DHF40-treated mice exhibited better beam walk performances with significantly reduced latency to cross the beam from 4 to 14 days after ICH (*P* < 0.01 at 4 days; *P* < 0.05 at 7 and 14 days; Fig. 1d). Significant differences in hindlimb score were also observed between the DHF40-treated and vehicle-treated groups at 4, 7, 21, and 28 days (*P* < 0.05 at 4 days; *P* < 0.01 at 7, 21, and 28 days; Fig. 1e). However, treatment with DHF20 only increased the rotarod running time at 14 days (Fig. 1c) and increased the beam score at 7 days (Fig. 1e), although beam walk latency was significantly shorter in the DHF20-treated group compared with the vehicle-treated groups from 1 day to 14 days after ICH (Fig. 1d). Taken together, these findings show that DHF40 can reduce long-term neurobehavioral deficits following ICH. We further investigated whether this treatment paradigm reduced brain edema, an important pathophysiological marker of secondary injury in ICH [26]. Brain water content, an indicator of brain edema, was significantly increased in the ipsilateral cortex and basal ganglia in the vehicle group compared with the contralateral counterpart (cortex: 79.9 ± 0.3% versus 78.3 ± 0.4%; *P* = 0.0071; basal ganglia: 84.5 ± 0.4% versus 77.9 ± 0.4%; *P* < 0.001) at 3 days post-ICH, which was significantly decreased with DHF40 treatment in the basal ganglia (82.2 ± 0. 5% versus 84.5 ± 0.4%, *P* = 0.005; Fig. 1f). We then assessed the safety of DHF40 in mice at 78 h post-ICH, at which point one dose of either vehicle or DHF40 was administered daily for 4 days. Treatment with DHF40 did not alter plasma concentrations of BUN and CRE, which are indicators of renal function, or ALT, an indicator of hepatic function (Table 1). Also, there were no differences in RBC, WBC and PLT counts or HGB levels among groups (Table 2). Similarly, no significant between-group differences were found in body weight change at 28 days (Fig. 1g) or in brain hemoglobin content, an indicator of hemorrhage size, at 3 days (Fig. 1h).

**Table 1:** Metabolic characteristics of the ICH mice treated with vehicle and 7,8-DHF (40 mg/kg)

	ICH Day 3		
	Vehicle (*n* = 6)	7,8-DHF (40 mg/kg) (*n* = 6)	Reference ranges [27]
BUN (mg/dL)	15.48 ± 1.92	15.27 ± 1.82	8–33
CRE (mg/dL)	0.23 ± 0.05	0.21 ± 0.03	0.2–0.9
ALT (IU/L)	30.83 ± 5.59	19.50 ± 1.72	17–77

Values are expressed as means ± SEM

*ICH* intracerebral hemorrhage, *DHF* 7,8-dihydroxyflavone, *BUN* blood urea nitrogen, *CRE* creatinine, *ALT* alanine aminotransferase

**Table 2:** Hematological characteristics of the ICH mice treated with vehicle and 7,8-DHF (40 mg/kg)

	ICH Day 3			
	Sham (*n* = 5)	Vehicle (*n* = 5)	7,8-DHF (40 mg/kg) (*n* = 5)	Reference ranges [28, 29]
RBC (10^6^/μL)	8.54 ± 0.40	8.40 ± 0.38	8.48 ± 0.34	8.46–9.81
HGB (g/dL)	13.94 ± 0.62	14.04 ± 0.36	14.07 ± 0.45	12.8–14.7
WBC (10^3^/μL)	3.98 ± 0.32	3.82 ± 0.13	4.09 ± 0.27	1.8–5.2
PLT (10^3^/μL)	736.60 ± 28.76	745.40 ± 53.53	766.00 ± 27.51	656–881

Values are expressed as means ± SEM

*RBC* red blood cell, *HGB* hemoglobin, *WBC* white blood cell, *PLT* platelet, *DHF* 7,8-dihydroxyflavone

### 7,8-DHF ameliorated brain tissue loss, neuronal damage and apoptosis in mice after ICH

**Fig. 2:**
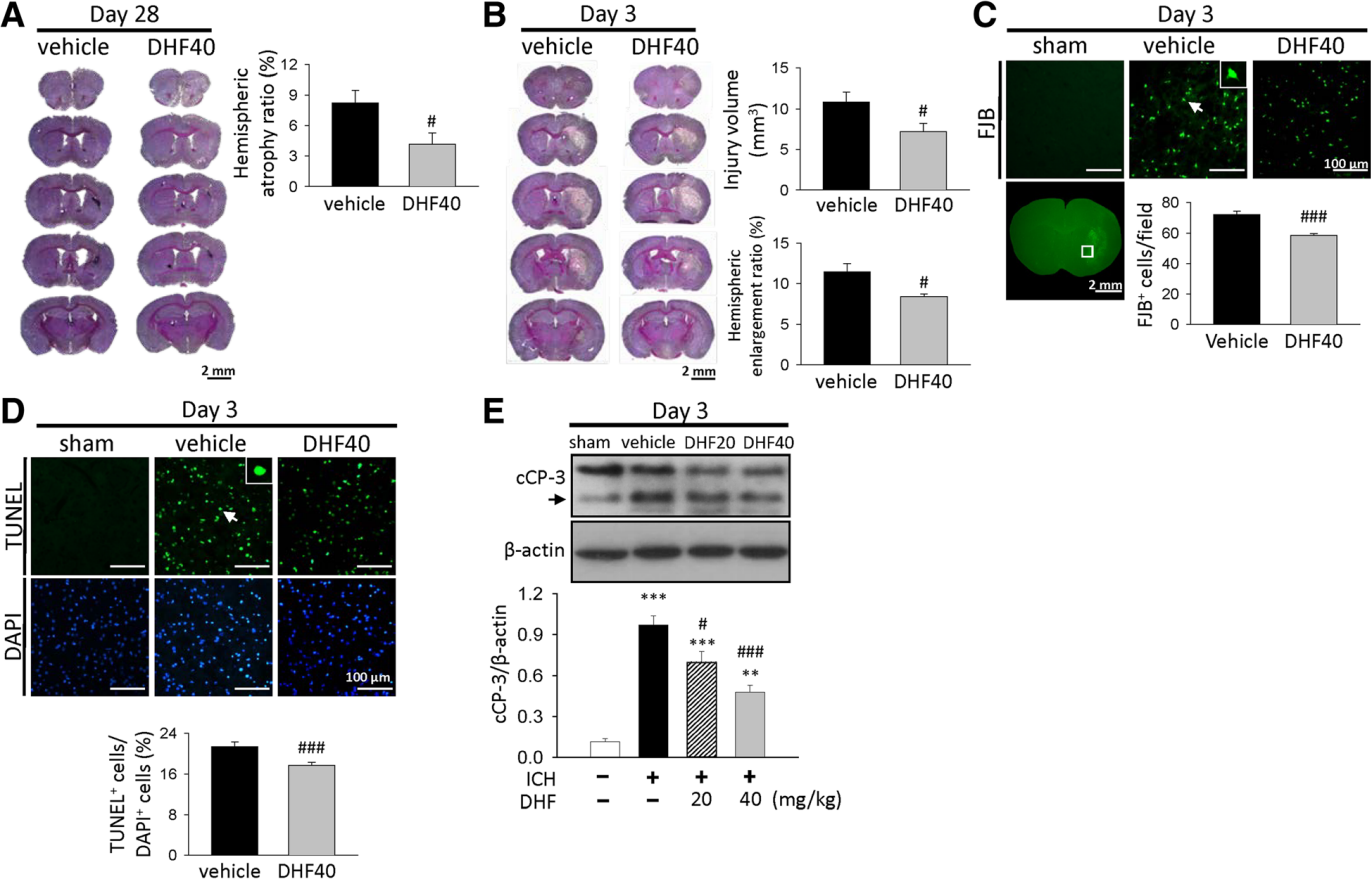
Activation of TrkB by 7,8-DHF treatment reduced brain tissue loss, neuronal damage and apoptosis after ICH. Representative cresyl violet-stained images and quantitative data of **a** brain atrophy ratio at 28 days; **b** hemorrhagic injury volume and hemispheric enlargement ratio at 3 day post-ICH. The scale bar is 2 mm. **c** Representative FJB-stained images and quantitative data of degenerating neurons at 3 days post-ICH. The inset is a representative FJB-positive cell at higher magnification. The scale bar is 100 μm. **d** Representative images of TUNEL (green) and DAPI (blue) stainings and quantitative data of apoptotic cells at 3 days post-ICH. The scale bar is 100 μm. **e** Representative immunoblots and quantitative data of cleaved caspase-3 levels were performed at 3 days after ICH. Values are mean ± S.E.M; ^**^P < 0.01 and ^***^P < 0.001 vs. sham group; ^#^*P* < 0.05 and ^###^*P* < 0.001 vs. vehicle group (*n* = 6 mice / group, Student’s t-test for histology and one-way ANOVA for Western blot). DHF20: 20 mg/kg 7,8-dihydroxyflavone; DHF40: 40 mg/kg 7,8-dihydroxyflavone; cCP-3: cleaved caspase-3

To determine whether the improvement in neurobehavioral function with 7,8-DHF is linked to a reduction in tissue damage and neuronal death, we next measured the extent of brain tissue damage and neuronal injury. ICH induced pronounced loss of tissue in the hemorrhagic hemisphere at 28 days post-ICH (Fig. 2a). However, DHF40 significantly reduced the degree of hemispheric atrophy compared to vehicle (4.2 ± 1.1% versus 8.3 ± 1.2%, *P* = 0.032; Fig. 2a) at 28 days. We further evaluated whether DHF40 attenuated brain tissue damage and neuronal injury during the acute stage of ICH. In keeping with the neuroprotective effect at 28 days, DHF40 treatment significantly reduced hemorrhagic injury volume to 66% of the vehicle group (7.2 ± 1.0 mm^3^ versus 10.9 ± 1.2 mm^3^, *P* = 0.041; Fig. 2b) at 3 days. Hemispheric enlargement, an indicator of brain edema, was also significantly smaller in DHF40-treated mice (8.4 ± 0.3%) than in vehicle-treated mice (11.5 ± 1.0%, *P* = 0.016; Fig. 2b) at 3 days. Similarly, the number of FJB-positive degenerative neurons around the hematoma was significantly decreased in DHF40-treated animals compared to vehicle-treated animals (58.7 ± 1.2 versus 72.0 ± 2.4 cells/field; *P* < 0.001; Fig. 2c). We next analyzed cell apoptosis after DHF40 treatment by TUNEL assay and Western blots for cleaved caspase-3, a critical effector caspase in apoptosis. TUNEL-positive nuclei were observed in the ipsilateral hemorrhagic hemisphere but not in the contralateral side at 3 days post-ICH; however, the DHF40-treated mice had significantly fewer TUNEL-positive cells around the hematoma than the vehicle group (17.7 ± 0.6% versus 21.3 ± 1.0%; *P* = 0.009; Fig. 2d). Similarly, cleaved caspase-3 was also reduced by 49.1% following DHF40 treatment compared to the vehicle group at 3 days post-ICH (*P* < 0.001; Fig. 2e).

### 7,8-DHF activated TrkB and downstream PI3K/Akt signaling, but did not affect Erk signaling after ICH

**Fig. 3:**
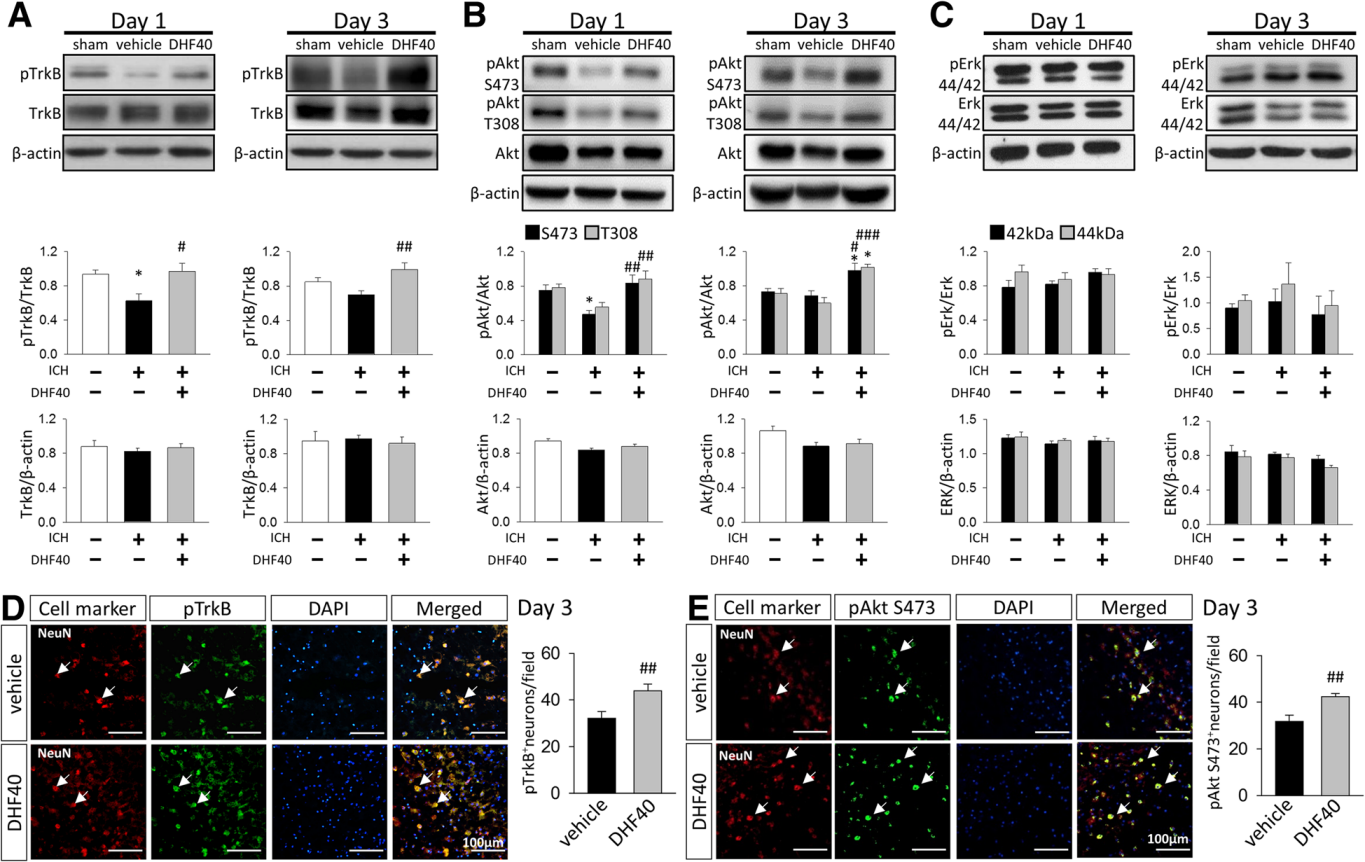
Activation of TrkB by 7,8-DHF treatment activated TrkB and downstream PI3K/Akt signaling, but did not affect Erk signaling after ICH. Representative immunoblots and quantitative data of **a** TrkB, **b** Akt and **c** Erk phosphorylation and total protein expression at 1 day and 3 days post-ICH. Values are mean ± S.E.M; ^*^P < 0.05 vs. sham group; ^#^*P* < 0.05, ^##^*P* < 0.01 and ^###^*P* < 0.001 vs. vehicle group (*n* = 6–7 mice / group, one-way ANOVA). Representative images of double immunostaining and quantitative data of **d** pTrkB-positive neurons and **e** pAkt Ser473-positive neurons in the peri-hematomal area at 3 days post-ICH. pTrkB or pAkt Ser473 is shown in green, and NeuN (neurons) is shown in red. Sections were stained with DAPI (blue) to show all nuclei. Values are mean ± S.E.M; ^##^*P* < 0.01 vs. vehicle group (*n* = 6–8 mice / group, Student’s t-test). DHF40: 40 mg/kg 7,8-dihydroxyflavone

We next sought to determine whether or not improved neurological function in 7,8-DHF-treated mice are associated with increased TrkB signaling in the hemorrhagic brain. To address this issue, we used western blot analysis to examine the phosphorylation of TrkB and its major downstream survival signaling pathways (the Akt and Erk pathways). ICH induced a significant decrease in the TrkB phosphorylation level at 1 day compared to sham-injury (*P* < 0.05). DHF40 significantly increased TrkB phosphorylation to 156% of its vehicle-level at 1 day (*P =* 0.017) and 142% at 3 days (*P =* 0.009, Fig. 3a). Likewise, levels of Akt Ser473 and Thr308 phosphorylation were significantly higher in the DHF40-treated group than in the vehicle-treated group at both 1 day (pAkt Ser473: 178% of vehicle group, *P =* 0.005; pAkt Thr308: 160% of vehicle group, *P =* 0.009; Fig. 3b) and 3 days (pAkt Ser473: 144% of vehicle group, *P* = 0.013; pAkt Thr308: 170% of vehicle group, *P =* 0.0003; Fig. 3b). In contrast, there was no significant effect of DHF40 treatment on the levels of Erk44/42 proteins at either time point (all *P* > 0.05; Fig. 3c). We also analyzed protein expression of total TrkB (Fig. 3a), total Akt (Fig. 3b) and total Erk (Fig. 3c) at both 1 day and 3 days after ICH, and results showed that there were no difference between groups (all *P* > 0.05). These findings demonstrate that 7,8-DHF induced TrkB activation and its downstream PI3K/Akt signaling following ICH.

We further analyzed whether 7,8-DHF affected pTrkB and pAkt Ser473 expression in neurons. An increase in the total number of pTrkB-positive neurons (*P =* 0.005; Fig. 3d) or pAkt Ser473-positive neurons (*P =* 0.005; Fig. 3e) was observed following 7,8-DHF treatment.

### 7,8-DHF enhanced the phosphorylation of Ask-1 Ser967 and FOXO-1 but did not affect the release of mitochondrial inner proteins into the cytosol

**Fig. 4:**
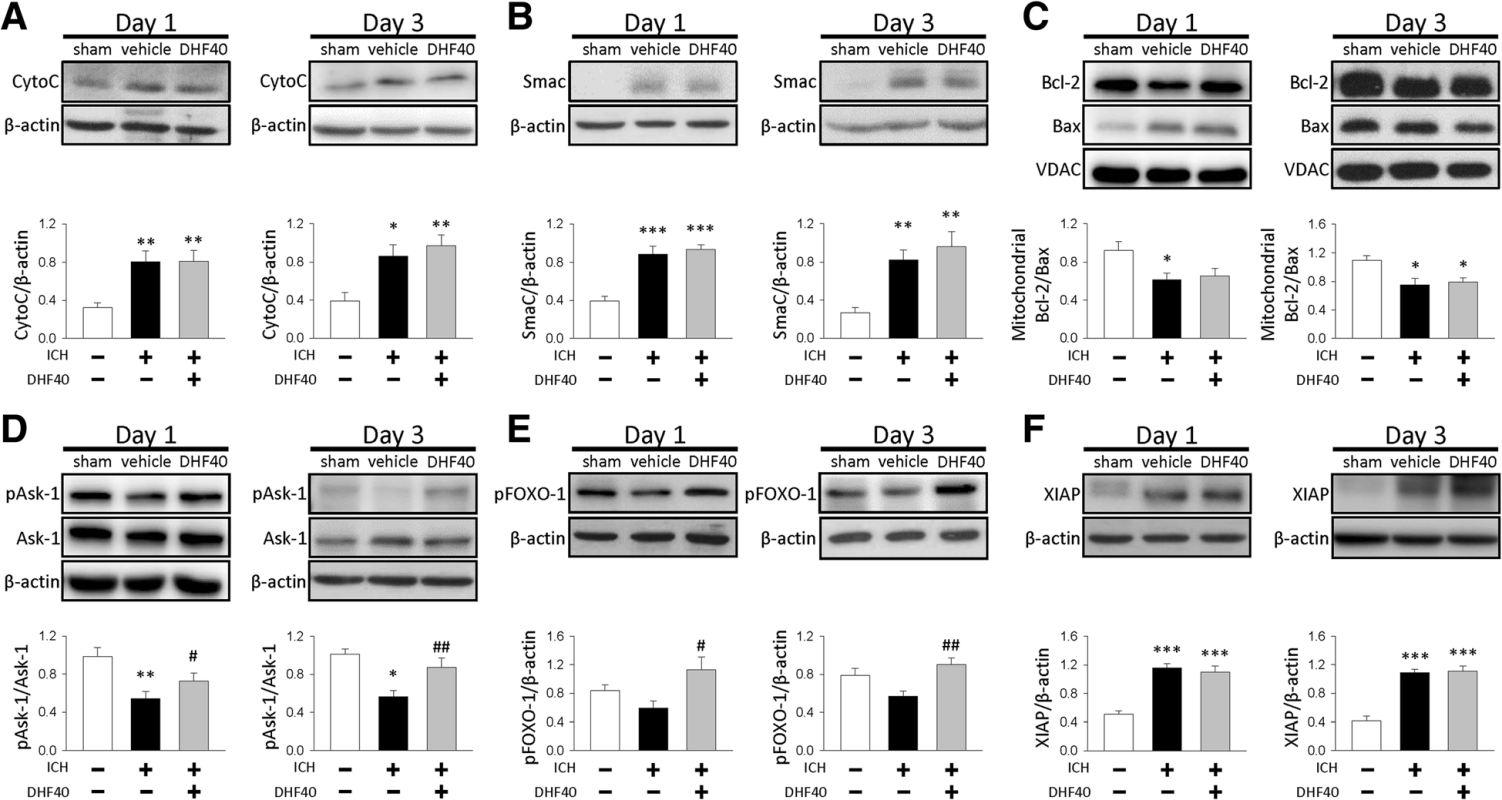
Activation of TrkB by 7,8-DHF treatment enhanced phosphorylation of Ask-1 Ser967 and FOXO-1 but did not affect the release of mitochondrial inner proteins into the cytosol after ICH. Representative immunoblots and quantitative data of **a** CytoC, **b** Smac/DIABLO, **c** mitochondrial Bcl-2 and Bax ratio, **d** Ask-1 Ser967 phosphorylation, **e** FOXO-1 phosphorylation and **f** XIAP levels at 1 day and 3 days post-ICH. Values are mean ± S.E.M; ^*^P < 0.05, ^**^P < 0.01 and ^***^P < 0.001 vs. sham group; ^#^*P* < 0.05 and ^##^*P* < 0.01 vs. vehicle group (*n* = 6 mice / group, one-way ANOVA). DHF40: 40 mg/kg 7,8-dihydroxyflavone

As DHF40 reduced ICH-induced caspase-3 activation, we next investigated whether DHF40 affected mitochondria-mediated apoptosis by interfering with the translocation of the mitochondrial inner proteins, CytoC and Smac/DIABLO, from the mitochondrial intermembrane space into the cytosol. We examined the amount of CytoC and Smac/DIABLO in the cytosolic fraction. In the sham-operated brains, only little or no CytoC and Smac/DIABLO was detected in the cytosol (Fig. 4a & b). Cytosolic CytoC and Smac/DIABLO were significantly increased in the vehicle group compared to the sham group at both 1 and 3 days post-ICH, suggesting that mitochondrial permeabilization was induced after ICH (all *P* < 0.05; Fig. 4a & b). However, DHF40 did not affect either cytosolic CytoC or Smac/DIABLO levels at both tested time-points. Given that the imbalance of proapoptotic Bcl-2 family proteins (e.g., Bax) and antiapoptotic Bcl-2 family proteins (e.g., Bcl-2) can be a primary cause of mitochondrial permeabilization, we also examined whether DHF40 treatment may reverse the ICH-induced imbalance of Bcl-2 and Bax, both of which are regulated by the PI3K-Akt survival pathway. We analyzed the ratio of Bcl-2 and Bax at the mitochondrial level. ICH induced a decrease in the Bcl-2/Bax ratio in the vehicle group at both 1 day and 3 days (both *P* < 0.05; Fig. 4c). However, no difference in the ratio following DHF40 treatment was observed. We further examined phosphorylation of Ask-1, FOXO-1 and XIAP, which are Akt downstream target molecules and are involved in regulating intrinsic or extrinsic apoptosis. Ask-1 is a MAP3K with proapoptotic activity principally through activation of the JNK or the p38 MAP kinase signaling cascades [30]. Dissociation of Ask-1 from the inhibitory protein 14–3-3 may result in Ask-1 activation. The phosphorylation of Ask-1 Ser967 enables Ask-1 to bind to 14–3-3, resulting in Ask-1 inhibition [31]. We then examined whether the extent of Ask-1 Ser967 phosphorylation was altered after DHF40 treatment. ICH induced a significant decrease in Ask-1 Ser967 phosphorylation in the vehicle group compared to the sham-injury group at both 1 day and 3 days (both *P* < 0.01). DHF40 significantly increased Ask-1 Ser967 phosphorylation to 133% of its vehicle-level at 1 day (*P* = 0.014) and 155% at 3 days (*P* = 0.0267, Fig. 4d). FOXO-1 is also a downstream target of Akt, promoting transcription of genes involved in both intrinsic and extrinsic apoptosis [32]. One mechanism by which Akt promotes cell survival is by phosphorylating FOXO-1, thereby inactivating it and preventing apoptosis. We thus studied the level of FOXO-1 phosphorylation by western blot analysis. Similar to the trend in Ask-1 Ser967 phosphorylation, the level of FOXO-1 phosphorylation was increased to 191% of the vehicle level at 1 day (*P* = 0.023) and 158% of the vehicle level at 3 days (*P* = 0.003) after DHF40 treatment (Fig. 4e). We also assessed the level of XIAP, which is a potent inhibitor of apoptosis by combining with caspase-3 and other members of the caspase family [11]. However, there was no difference in the XIAP level between the vehicle-treated and DHF40-treated groups. Taken together, these results indicate that DHF40 enhanced the phosphorylation of Ask-1 Ser967 and FOXO-1 but did not affect the release of mitochondrial inner proteins into the cytosol.

### 7,8-DHF attenuated neuronal death in the in vitro ICH model

**Fig. 5:**
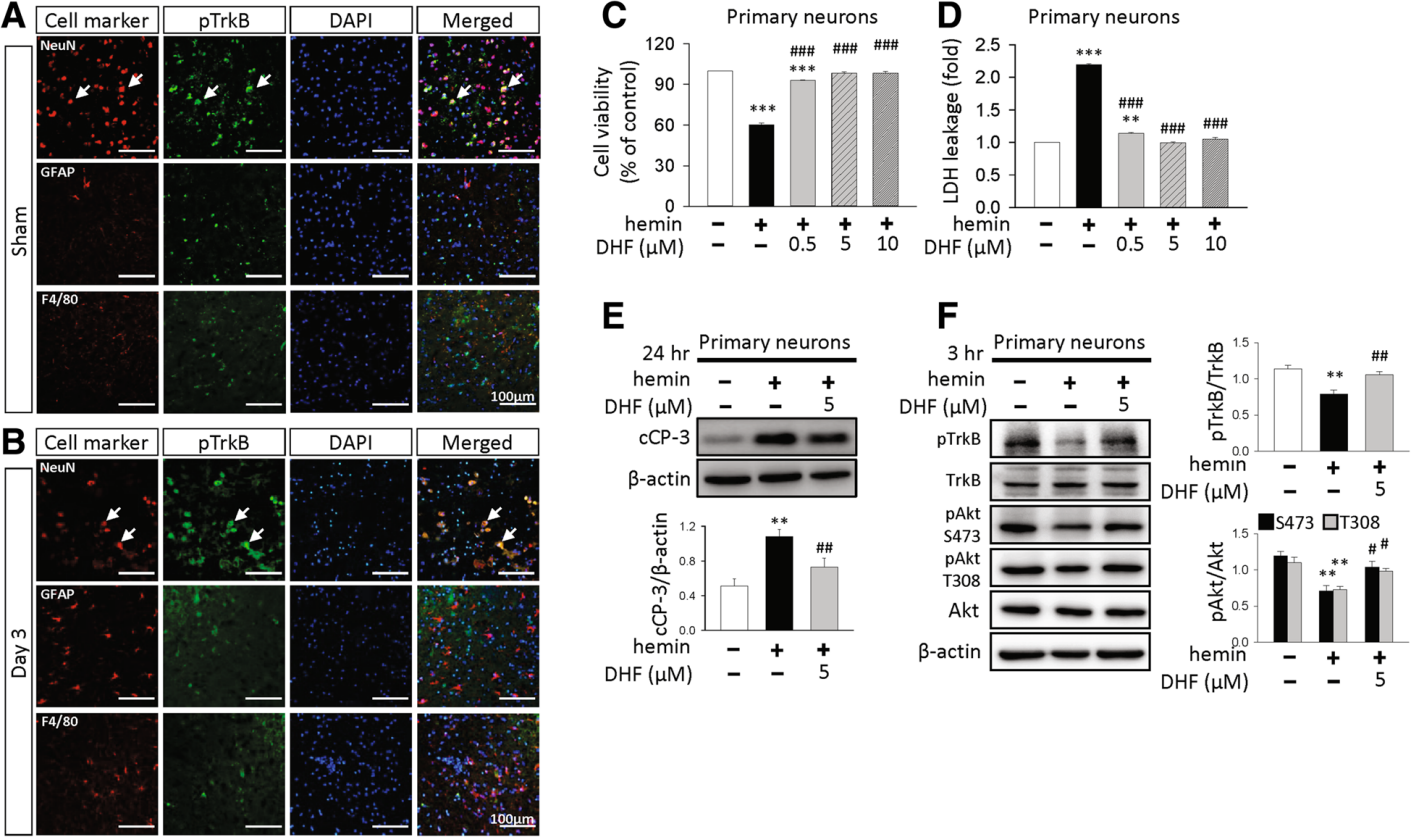
Activation of TrkB by 7,8-DHF treatment attenuated neuronal apoptosis in vitro and increased BDNF expression after ICH. Representative images of double immunostaining for pTrkB (green) and neurons (NeuN), astrocytes (GFAP) or microglia (F4/80) (red) in **a** sham group or in **b** the peri-hematomal area at 3 days post-ICH. Yellow labeling (white arrows) indicates co-localization. Sections were stained with DAPI (blue) to show all nuclei. The scale bar is 100 μm. **c** Cell viability assessed by MTT assay, **d** cytotoxicity assessed by LDH assay and **e** representative immunoblots and quantitative data of cleaved caspase-3 expression in primary cultured neurons stimulated with hemin for 24 h. **f** Representative immunoblots and quantitative data of TrkB and Akt phosphorylation in primary cultured neurons stimulated with hemin for 3 h. Values are mean ± S.E.M; ^**^P < 0.01 and ^***^P < 0.001 vs. control group; ^#^*P* < 0.05, ^##^*P* < 0.01 and ^###^*P* < 0.001 vs. hemin-treated group (*n* = 4–5 independent experiments/group, one-way ANOVA). DHF40: 40 mg/kg 7,8-dihydroxyflavone; cCP-3: cleaved caspase-3

To validate that TrkB activation induced by 7,8-DHF directly occurred in neurons, we examined the localization of the pTrkB following ICH by double immunofluorescence. TrkB phosphorylation was found mainly in neurons in the striatum of the sham group (Fig. 5a) or around the hematoma at 3 days following ICH (Fig. 5b), but rarely in astrocytes or microglia.

We next assessed the impact of 7,8-DHF in the in vitro ICH model. Lysis of red blood cells after ICH causes release of hemoglobin, which is further broken down into heme or its oxidized form hemin. Hemin has been used in vitro to study ICH-induced neuronal death in primary neurons [23]. Incubation of primary neurons with 20 μM hemin for 24 h led to a significant decrease in cell viability (60.5 ± 1.1% compared to that of control, Fig. 5c). Cotreatment with 0.5 μM, 5 μM, or 10 μM 7,8-DHF increased cell viability to 92.8, 98.3, and 98.3%, respectively of the control-level (all *P* < 0.001, Fig. 5c). Likewise, exposure of primary neurons to 20 μM hemin significantly increased LDH release, an indicator of cellular injury, to 219.4% of the control level. Cotreatment with 0.5 μM, 5 μM, or 10 μM 7,8-DHF reduced LDH release to 114.5, 99.8, and 104.8%, respectively of the control-level (all *P* < 0.001; Fig. 5d) and 5 μM provided the best protection. In addition, the cleaved caspase-3 level in primary neurons treated with 5 μM 7,8-DHF was also significantly decreased to 67.8% (*P* = 0.008) of the control-level (Fig. 5e).

We further determined whether improved survival in 7,8-DHF-treated primary neurons are associated with increased TrkB and PI3K/Akt activation. Hemin treatment for 3 h induced a significant reduction of TrkB phosphorylation, Akt phosphorylation at both Ser473 and Thr308 compared to the control group (all *P* < 0.05; Fig. 5f). Similar to the results in Fig. 3, 7,8-DHF treatment reversed the reduction of TrkB phosphorylation, pAkt Ser473 and pAkt Thr308 phosphorylation (all *P* < 0.05; Fig. 5f). Taken together, these results indicate that 7,8-DHF is directly protective against hemin-induced neuronal damage.

**Fig. 6:**
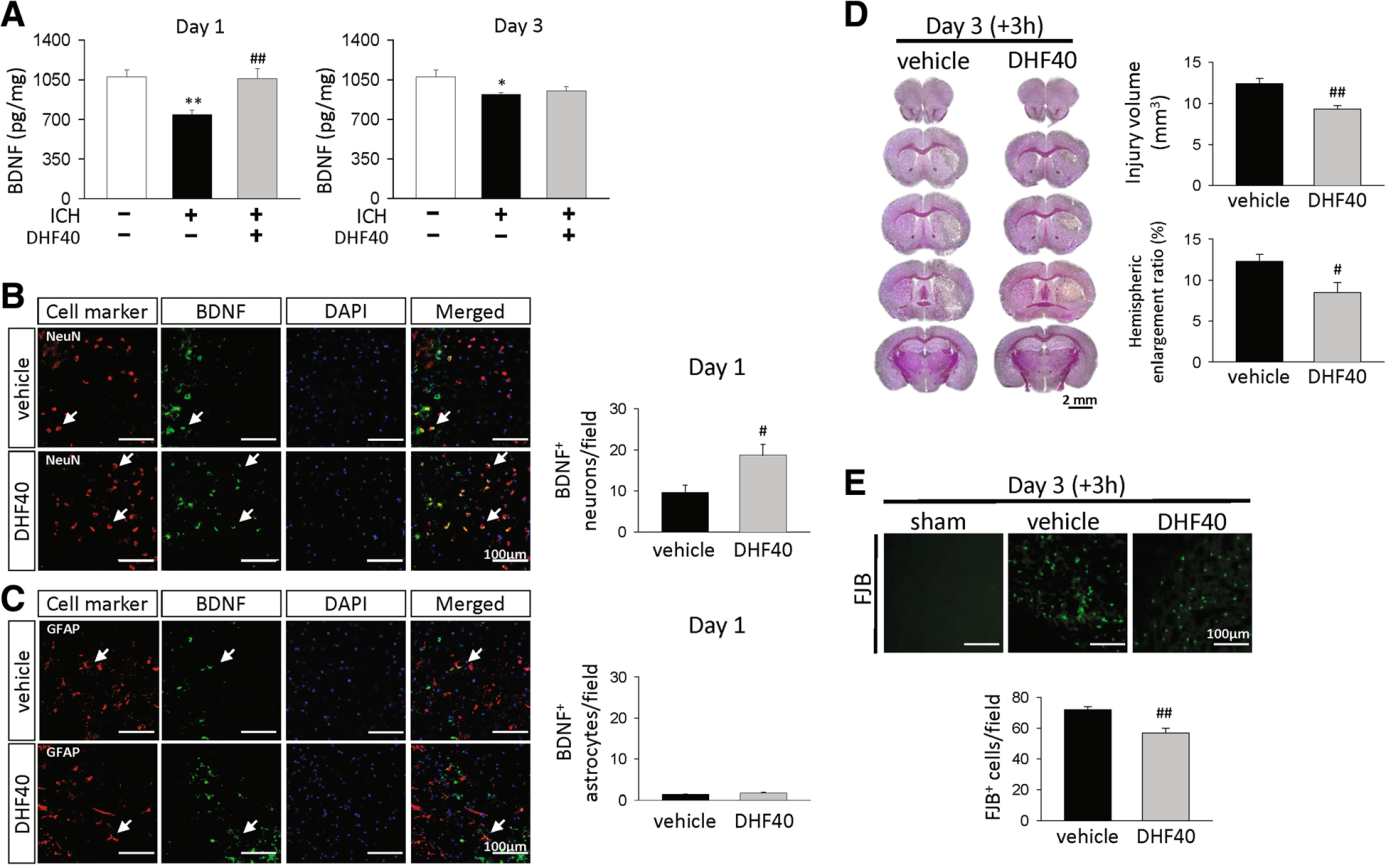
Delayed post-injury administration of 7,8-DHF reduced brain damage and neuronal death after ICH. **a** BDNF protein levels measured by ELISA at 1 day and 3 days post-ICH. Values are mean ± S.E.M; ^*^P < 0.05 and ^**^P < 0.01 vs. sham group; ^##^*P* < 0.01 vs. vehicle group (*n* = 5–7 mice/group, one-way ANOVA). Representative images of double immunostaining and quantitative data of **b** BDNF-positive neurons (NeuN) and **c** BDNF-positive astrocyte (GFAP) at 1 day post-ICH. BDNF is shown in green, and NeuN or GFAP is shown in red. Values are mean ± S.E.M; ^#^*P* < 0.05 vs. vehicle group (*n* = 6 mice / group, Student’s t-test). Treatment of 7,8-DHF was initiated at 3 h after ICH induction. **d** Representative cresyl violet-stained images and quantitative data of hemorrhagic injury volume and hemispheric enlargement ratio at 3 days post-ICH. The scale bar is 2 mm. **e** Representative FJB-stained images and quantitative data of degenerating neurons at 3 days post-ICH. Values are mean ± S.E.M; ^#^*P* < 0.05 and ^##^*P* < 0.01 vs. vehicle group (*n* = 6 mice / group, Student’s t-test). DHF40: 40 mg/kg 7,8-dihydroxyflavone, + 3 h: treatment initiated at 3 h post-ICH

Previous studies have shown that BDNF/TrkB signaling can self-amplify BDNF actions through positive feedback mechanisms [33]. To determine whether 7,8-DHF would trigger further BDNF production, we measured the brain levels of BDNF. The BDNF protein levels were significantly decreased in the ipsilateral hemisphere at both 1 day and 3 days after ICH compared with sham-injury (both *P* < 0.05; Fig. 6a). DHF40 substantially increased BDNF to 143% of the vehicle-level in the ipsilateral hemisphere (*P* = 0.004; Fig. 6a). However, although the BDNF level was slightly increased following DHF40 treatment at 3 days, it did not reach statistical significance. To validate that BDNF induced by 7,8-DHF directly occurred in neurons or astrocyte. We measured the brain levels of BDNF. The BDNF protein levels after 7,8-DHF treatment were significantly increased in neurons (*P =* 0.018; Fig. 6b) but not astrocyte (*P* > 0.05; Fig. 6c) at 1 day after ICH compared to vehicle group.

### Delayed post-injury administration of 7,8-DHF reduced brain damage and neuronal death

To determine whether 7,8-DHF could still exert beneficial effects when administered at later time points after ICH, 7,8-DHF treatment was delayed by 3 h post-injury. Interestingly, 7,8-DHF treatment initiated at 3 h post-ICH still significantly reduced injury volume (9.3 ± 0.4 mm^3^ versus 12.4 ± 0.7 mm^3^; *P* = 0.002; Fig. 6d) and hemispheric enlargement, an indicator of brain edema, (8.5 ± 1.2% versus 12.3 ± 0.9%, *P* = 0.0302; Fig. 6d) at 3 days. The number of FJB-positive neurons around the peri-hematoma area was also reduced (56.7 ± 3.2 versus 72.0 ± 2.0 cells/field; *P* = 0.025; Fig. 6e) compared to vehicle at 3 days post-injury. The neuroprotective effects of DHF40 either initiated immediately or after a 3 h delay following ICH were comparable; injury volume, hemispheric enlargement and neuronal damage were attenuated by 34, 27 and 19%, respectively when administered immediately post-ICH (Fig. 4) and by 24, 31 and 21%, respectively when treatment was started at 3 h post-injury (Fig. 6). These results suggest a therapeutic window of 7,8-DHF treatment for ICH, at least up to 3 h.

## Discussion

This study demonstrated for the first time that post-injury 7,8-DHF treatment attenuated brain tissue damage, brain edema and long-term behavioral deficits following ICH in mice, and decreased neuronal vulnerability to hemin-induced injury in vitro. Moreover, 7,8-DHF also suppressed apoptosis and neuronal damage both in vivo and in vitro. These effects engaged molecular systems related to the activation of the TrkB-PI3K/Akt pathway and enhanced the phosphorylation of Akt downstream molecules, Ask-1 and FOXO-1. However, the release of mitochondrial inner proteins into the cytosol was not affected following 7,8-DHF treatment. We further showed that 7,8-DHF also exerted significant protective effects using a more clinically relevant treatment time window (3 h post-ICH). Our results indicate that enhancing TrkB activation may provide a potential therapy for ICH.

We found that systemic administration of 7,8-DHF for 4 days significantly reduced brain atrophy and enhanced functional improvement up to 28 days after ICH. This protective effect was accompanied by a decrease in neuronal damage and apoptotic cell death, and a reduction in brain edema at day 3. As apoptosis is the predominant form of cell death around the hematoma following ICH [34], the long-term protective effect in functional recovery with 7,8-DHF treatment is possibly due to a reduction in the number of apoptotic neurons detected during the acute phase and the attenuation of long-term tissue injury. Our findings suggest that 7,8-DHF effectively ameliorated the pathological events leading to ICH-induced neuronal damage during the first 3 days, which consequently led to a better prolonged recovery of neurological function. This improvement in long-term functional outcomes is of particular importance as only 20% of ICH patients eventually regain functional independence [35].

Most previous reports have investigated BDNF expression following ICH. In this study, we extended the previous findings by examining both BDNF expression and BDNF signaling activity, by assessing TrkB phosphorylation and its downstream Akt and Erk signals. We showed that 7,8-DHF increased TrkB and Akt activation at the acute stage, which was paralleled by a reduction of functional and histological deficits over 1 month. The number of pTrkB-positive neurons and BDNF protein levels also increased following 7,8-DHF treatment. In the in vitro ICH model, 7,8-DHF promoted neuronal survival and reduced apoptosis in primary cultured neurons exposed to hemin. These results suggest that BDNF/TrkB signaling is involved in regulating neuronal survival after ICH. Consistent with our findings, several preclinical studies, using various therapeutic interventions, such as endothelial progenitor cells transplantation [36], hydrogen inhalation [15], and rehabilitative training [37], have reported BDNF signaling as a potential therapeutic target following ICH. Together with previous reports, our findings support the speculation that BDNF-TrkB signaling participates in the pathophysiological process of ICH.

We observed that 7,8-DHF increased BDNF protein expression in neurons but not in astrocytes at 1 day after ICH. Another study in experimental cerebral ischemia has also found that neurons but not astrocytes expressed BDNF mRNA in the ischemic striatum [38]. Our previous study in experimental traumatic brain injury further showed that 7,8-DHF application induced BDNF mRNA upregulation through enhancing CREB activation in neurons [19]. Together, these results suggest that neurons could be the main source of BDNF following brain damage.

We demonstrated that 7,8-DHF increased Akt phosphorylation at both Ser473 and Thr308 residues, without affecting Erk44/42 phosphorylation following mouse ICH. The current results are in lines with previous studies in mouse traumatic brain injury [19] and in the 6-hydroxydopamine-induced neuronal death model [39] showing that 7,8-DHF increased Akt but did not alter Erk44/42 phosphorylation. Although Erk44/42 activation has generally been associated with brain cell differentiation and proliferation, a number of studies have shown that the activation of Erk44/42 can mediate cell death in various neurological disease models [40]. The activation of Erk44/42 was observed in glutamate- and heme-induced neuronal cell death [41, 42] and neuronal injury was reduced when Erk44/42 activation was suppressed in thrombin-induced brain damage [43]. In contrast, accumulating evidence has demonstrated that the PI3K/Akt is a major survival pathway in various neurological disorders [44, 45]. Akt regulates apoptosis, either by transcription or direct phosphorylation. Activated Akt phosphorylates the death promoter Bad to maintain mitochondrial integrity by preventing the inhibition of anti-apoptotic Bcl-2 by Bad [13]. Activated Akt also blocks Fas ligand transcription by phosphorylating FOXO, thereby interfering with ligand-induced extrinsic apoptosis [14]. Furthermore, Akt enhanced the phosphorylation of Ask-1 Ser967, which is known to promote the association between Ask-1 and 14–3-3 protein, and suppress JNK and c-Jun activation, ultimately inhibiting cellular apoptosis. Taken together, our results suggest that Akt and Erk pathways may play different roles in mediating neuronal survival. Our data underscore the importance of defining survival pathways that counteract apoptosis induced during ICH. Nevertheless, we cannot exclude the possibility that 7,8-DHF administration may have effects on ICH-induced inflammatory responses as previous studies have shown that 7,8-DHF administration attenuated astrogliosis in mice after perinatal hypoxia and ischemia [46] and reduced the release of pro-inflammatory mediators in lipopolysaccharide-stimulated BV2 microglial cells [47]. Further investigations are needed to clarify the anti-inflammatory mechanism underlying the 7,8-DHF-mediated neuroprotection in ICH.

We showed that 7,8-DHF administration did not affect the release of mitochondrial inner proteins into the cytosol or the mitochondrial Bcl-2/Bax ratio at 1 and 3 days post-ICH, despite a reduction in cleaved caspase-3 level following 7,8-DHF treatment compared to the vehicle group at 3 days. The results are different from our previous work in mouse traumatic brain injury showing that 7,8-DHF administration increased the Bcl-2/Bax ratio at 4 days post-TBI [19]. One possible explanation for this disparity may be due to different pathology from disease to disease. Indeed, different types of primary insults may result in diverse cellular vulnerability patterns as well as a spectrum of injury processes [48]. For example, TBI involves a primary mechanical impact which triggers release of glutamate and calcium influx [49]. This large, sustained influx of calcium into cells can initiate many intracellular pathways and induce the intrinsic apoptotic pathway [49]. However, in ICH, the inflammatory responses triggered by injured neuronal cells and blood components such as thrombin, fibrin and heme have been regarded as a major pathological event [50]. These inflammatory products could activate death receptors and subsequently causes the extrinsic apoptotic pathway [51]. Thus, it is possible that 7,8-DHF have different protective effects on ICH and TBI. Another possibility is the time points examined. Although we did not observe changes of CytoC and Smac/DIABLO release from mitochondria following 7,8-DHF administration at 1 day and 3 days after ICH, this does not exclude the possibility that 7,8-DHF administration could be protective against mitochondrial apoptosis at later time points following ICH as different disease processes may have different therapeutic time windows. Also, the brain lysate contains not only neurons but also glial cells, and ICH-induced glial activation and 7,8-DHF effect would also affect the mitochondrial signaling directly and indirectly. Indeed, BDNF protected astrocytes from cell death and induced release of neuroprotective factors from astrocytes [52], suggesting that 7,8-DHF might have effects on the mitochondrial signaling of astrocytes.

In the current study, Ask-1 Ser967 and pFOXO-1 was found to increase following 7,8-DHF treatment. Increased Akt activity can lead to phosphorylation of Ask-1 and FOXO-1, which may be the underlying mechanism for the prosurvival effects of Akt via regulating extrinsic apoptosis. FOXO-1 directly regulates the extrinsic apoptotic pathway through stimulating expression of death receptor ligands such as Fas ligand and tumor necrosis factor-related apoptosis-inducing ligand (TRAIL) [10]. Ask-1 is a member of the MAP3K family that induces apoptosis via activating downstream MAPKs, JNK and p38 MAPKs [9]. Activated Ask-1 induces the activation of its downstream targets JNK and p38 kinase, which induces apoptosis in neurons in vitro and in the brain [53]. Previous in vitro studies have demonstrated that Ask-1 promotes apoptosis via activation of caspase-8. Results of our study, using brains affected by ICH highlight a remarkable decrease in Ask-1 Ser967 and FOXO-1 phosphorylation in the hemorrhagic hemispheres, and the restoration of phosphorylation levels by 7,8-DHF. These findings confirm results of previous studies, showing that genetic knockdown or pharmacological inhibition of Ask-1 or FOXO-1 reduced brain damage after brain damage caused by cerebral ischemia and traumatic brain injury [54–56], suggesting that Ask-1 and FOXO-1 are potential therapeutic targets in ICH. However, further investigations are needed to clarify the mechanism underlying the anti-apoptotic mechanisms following 7,8-DHF treatment in ICH.

In this study, mice received a relative high (60% in 0.1 ml) but non-toxic dose of DMSO when treated with 7,8-DHF [57]. As a new type of non-viral vector, liposomes have been shown to carry BDNF across the blood-brain barrier and into the brain [58, 59]. Clearly, liposomal formulations to enhance 7,8-DHF delivery to the brain warrants further investigation.

## Conclusions

**Fig. 7:**
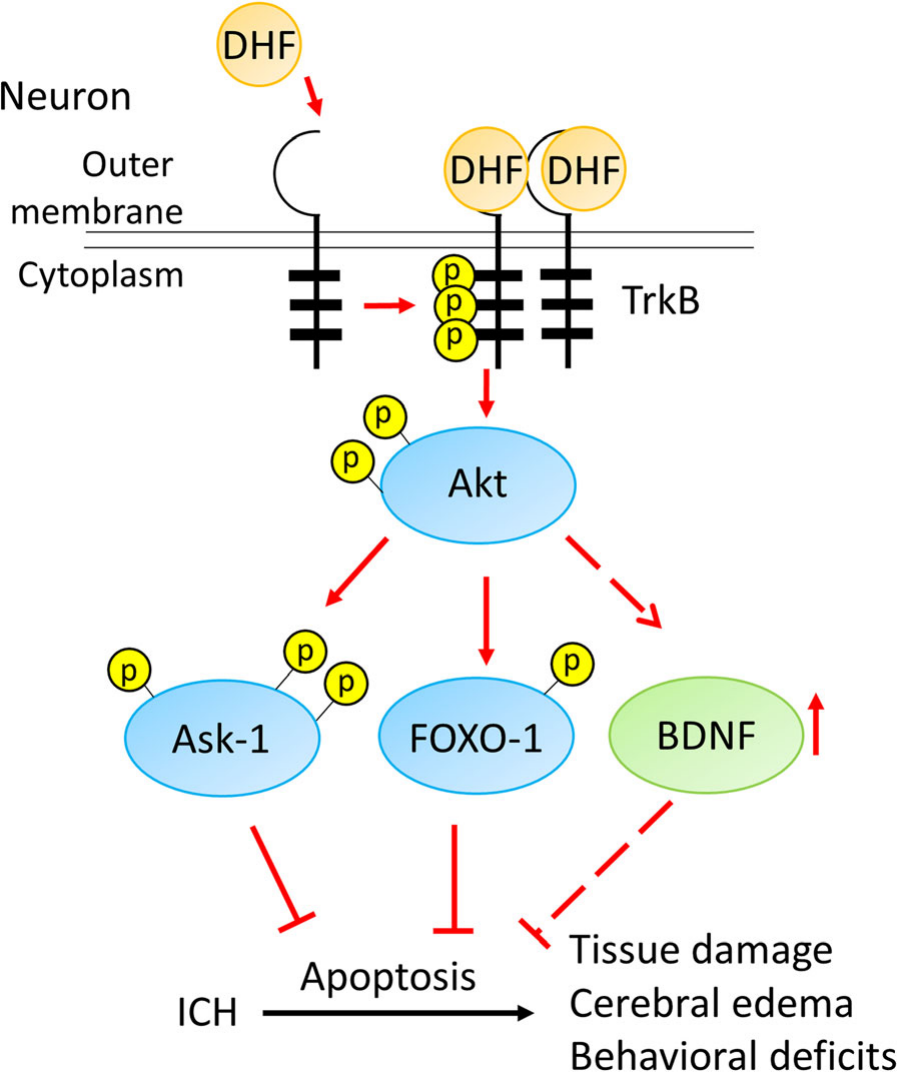
The schematic diagram indicates the potential mechanisms of TrkB activation-induced neuroprotection in ICH mice. As summarized in Fig. 7, we found that treatment with 7,8-DHF promoted neuronal survival and reduced apoptosis, attenuated brain tissue damage, cerebral edema and behavioral deficits following ICH. These effects were related to increase TrkB and subsequently Akt activation in neurons, and enhancing Ask-1 and FOXO-1 phosphorylation after ICH. 7,8-DHF also increased short-term BDNF expression after ICH. Our findings suggest that pharmacological enhancement of TrkB signaling by 7,8-DHF could be a potential strategy for the management of ICH. DHF: 7,8-dihydroxyflavone

As summarized in Fig. 7, we found that treatment with 7,8-DHF promoted neuronal survival and reduced apoptosis, attenuated brain tissue damage, cerebral edema and behavioral deficits following ICH. These effects were related to increased TrkB and subsequently Akt activation in neurons, and enhancing Ask-1 and FOXO-1 phosphorylation after ICH. 7,8-DHF also increased short-term BDNF expression after ICH. Our findings suggest that pharmacological enhancement of TrkB signaling by 7,8-DHF could be a potential strategy for the management of ICH.

## Data Availability

All data used during the current study available from the corresponding author on reasonable request.

## Abbreviations

7,8-DHF: 7,8-dihydroxyflavone
ALT: Alanine aminotransferase
ANOVA: Analysis of variance
Ask-1: Apoptosis signal-regulating kinase 1
BBB: Blood-brain barrier
BDNF: Brain-derived neurotrophic factor
BUN: Blood urea nitrogen
CRE: Creatinine
CytoC: Cytochrome C
FJB: Fluoro-Jade B
HGB: hemoglobin
ICH: Intracerebral hemorrhage
Ip: Intraperitoneally
JNKs: c-Jun N-terminal kinases
LDH: Lactate dehydrogenase
MAP3K: Mitogen-activated protein kinase kinase kinase
MAPKs: MAP kinases
mNSS: Modified neurological severity score
MTT: 3-[4,5-dimethyl-2-thiazolyl]-2,5-diphenyl-2-tetrazolium bromide
PI3K: Phosphatidylinositol 3-kinase
PLT: Platelet
RBC: Red blood cell
SEM: Standard error of the mean
TdT: Terminal deoxynucleotidyl transferase
TNF: Tumor necrosis factor
TrkB: Tropomyosin-related kinase receptor B
TUNEL: Terminal deoxynucleotidyl transferase dUTP nick end labeling
WBC: White blood cell
XIAP: X-linked inhibitor of apoptosis protein
